# Attachment as the Catalyst for the Attribution of Complex Cognition and Emotion to Companion Cats

**DOI:** 10.3390/ani14142123

**Published:** 2024-07-21

**Authors:** Jennifer Vonk, Esther M. C. Bouma

**Affiliations:** 1 Department of Psychology, Oakland University, Rochester, MI 48363, USA; 2 Department of Social Psychology, Faculty of Behavioural and Social Sciences, University of Groningen, P.O. Box 72, 9700 AB Groningen, The Netherlands; e.m.c.bouma@rug.nl

**Keywords:** domestic cats, primary emotions, secondary emotions, caregiver bonds, housing

## Abstract

**Simple Summary:**

Companion animal caregivers who have stronger attachment bonds to their companion animals ascribe more complex emotions and cognitions to them compared to those with lower levels of attachment. We examined whether the experience of caring for cats, caregiver attachment, and style of attachment (avoidant, anxious) predicted greater attributions of primary and secondary emotions and cognitive abilities to companion cats. Those with avoidant attachment styles perceived fewer primary emotions in their cats, whereas those with stronger attachment strength perceived their cats to express more secondary emotions and cognitive capacities. Both cat-caregivers and non-cat-caregivers with greater beliefs in animal minds perceived more complex secondary emotions and cognition. Attitudes toward animals predicted only the attribution of primary emotions, and housing cats indoors or outdoors did not uniquely predict attributions. As with previous studies, the type and strength of the bond formed with companion cats is strongly associated with the way caregivers perceive their thoughts and feelings, which likely impacts feline welfare. Our findings suggest that our general liking for animals is less important than our bonds with specific animals in shaping our thoughts about their minds.

**Abstract:**

Companion cat caregivers ascribe complex emotions and cognitions to their cats, and these attributions are greater with a stronger attachment to their animals. We compared attributions of emotional and cognitive complexity to cats in cat caregivers and non-caregivers. We measured attitudes toward animals, belief in animal minds, caregivers’ strength of attachment, and attachment style with a particular companion cat in 448 university students, of whom 251 had owned a cat. We asked the extent to which respondents thought that cats were capable of expressing primary and secondary emotions and cognitive capabilities. Caregivers significantly differed from non-caregivers only in the attribution of primary emotions. Belief in animal minds predicted the attribution of secondary emotions and cognition. For caregivers only, avoidant attachment style was negatively associated with the attribution of primary emotions, whereas attachment strength was positively associated with the attribution of secondary emotions and cognition. These abilities may have greater discriminatory power as most respondents ascribe primary emotions to cats, perhaps for anthropomorphic reasons. Housing conditions (indoor and outdoor) were not associated with attributions, suggesting that bonds are more important than time spent together within the home in predicting the attribution of emotional and cognitive complexity in cats.

## 1. Introduction

Previous studies have established that pet owners attribute greater emotional [1,2,3,4,5] and cognitive [1,6] complexity to their companion animals when they have stronger attachments to these animals. Most prior studies focused on the role of the companion animal (i.e., as family member versus pet) or overall strength of attachment to the animals but did not examine specific attachment styles, such as avoidant and anxious attachments [7]. Interestingly, these insecure attachment styles predict empathy toward animals [8] and, when applied to companion animal relationships [9], predict the likelihood to of adoption [10] as well as surrender of companion animals [11]. Individuals with avoidant attachment to deceased companion animals attribute less grief to surviving animals [12]. One of the few other studies to explore attachment styles in relation to the tendency to attribute human-like characteristics to animals found that avoidant attachment styles were negatively associated and anxious attachment styles were positively associated with anthropomorphism [13]. Owners have previously described relationships with cats as more consistent with avoidant attachment, whereas they described relationships with dogs as akin to secure attachment bonds [10,13]. These studies suggest that it is important to consider attachment style in addition to attachment strength, particularly when comparing owner attachments between cats and dogs. We examined whether avoidant or anxious attachment styles, in addition to attachment strength, were associated with greater attribution of emotional and cognitive complexity to domestic cats, given that these relationships are less well understood compared to dog–caregiver relationships.

In line with previous research [3,4,5,14], we separately examined the attribution of primary and secondary emotions. It is thought that primary emotions, such as fear and anger, are more or less innate and spontaneously elicited, whereas secondary emotions (e.g., shame, pride, guilt, jealousy, and embarrassment) may reflect self-consciousness and involve greater reflection. Whereas primary emotions are thought to be widely experienced across the animal kingdom, the existence of secondary emotions in non-primate species is contested [3,15,16]. Morris et al. [3] found that most survey respondents ascribed primary emotions to their companion animals, but almost half of dog, cat, and horse owners also ascribed secondary emotions like pride and shame to their animals. 

Companion animal caregivers ascribe complex emotions and abilities more readily to dogs compared to other species [1,6,13,14,17,18], although [5] found no difference in the ascription of emotion between dogs and cats, except that anger was more frequently ascribed to dogs. Similar behavioral changes to the loss of a companion animal have been reported in cats and dogs, suggesting that if these animals grieve, they may do so similarly [12,19]. Arahori et al. [17] found that dogs were described as more likely to “have” sadness, friendship, sympathy, compassion, and pity compared to cats; however, no differences were found for jealousy or hatred. Pickersgill et al. [14] also found that caregivers believed that dogs could express more emotions than cats could. Interestingly, caregivers who cared for both dogs and cats attributed fewer emotions to cats compared to caregivers who cared for cats alone, suggesting that direct experience with both dogs and cats in the home may highlight differences in emotional range due to contrast effects. It is also possible that humans are better able to accurately recognize emotions in dogs compared to cats, a hypothesis that would be difficult but important to examine. Notably, some researchers have suggested that dog owners can accurately perceive at least some dog emotions [20,21,22], whereas other research suggests that owners may misattribute emotions in cats [2]. Further research regarding the expression of emotion in cats is warranted. 

Whereas previous studies examined the expression of emotions in companion animals, most did not delve into how well animals understand emotion states by attributing emotion states to others. One exception, Arahori et al. [17] found that dogs were rated by their caregivers as being better able to understand human attentional states compared to cats, whereas caregivers reported no difference in dogs’ and cats’ abilities to reason or read human intentions. Bouma et al. [1] also asked whether cats and dogs understood human emotions and intentions but did not report separate results for these items, which were part of their general “comparative anthropomorphization (COANT)” measure. These authors did not analyze whether differences in attachment strength or style predicted these differences in attribution of emotion and intellectual abilities. Vonk [6] examined differences in abilities ascribed to cats and dogs as a function of caregiver attachment strength and found that, in general, the stronger the attachment bond, the more both cat and dog caregivers attributed positive attributes and greater cognitive abilities. This study included questions about a range of cognitive capacities rather than expression of emotion alone and also found that caregivers attributed greater abilities to dogs compared to cats, except with regard to hunting/exploration, for which they rated cats as being more skilled. In the current study, we assessed various aspects of cognition to broaden our understanding of caregiver perceptions. 

Most relevant research has focused exclusively on the attributions of companion animal caregivers rather than comparing the attributions of caregivers to those of non-caregivers. An exception found that companion animal caregivers were more likely to believe that their animals had emotional experiences compared to non-caregivers [23]. It is unknown whether these differences are due simply to the experience of caring for and observing companion animals within the household or due to the formation of a strong emotional bond, which may lead to a greater tendency to anthropomorphize [1] or greater empathy for the companion animal [24,25,26], which predicts greater attribution of emotion [27,28]. Although one cannot examine whether potential differences between cat caregivers and non-caregivers are mediated by attachment to companion animals (which would be unavailable in non-caregivers), we compared their attributions using a measure of attitudes toward pets [29], which is typically highly correlated with attachment for pet owners [30], and a general measure of belief in animal minds [28]. Thus, we could examine whether, compared to non-cat caregivers, cat caregivers were more likely to attribute more complex emotions and cognitions to their companion cats and whether these attributions were associated with greater attachment to their own companion, specifically or attitudes toward animals generally. 

Other aspects of animal–caregiver relationships, such as the role of the animal in the caregiver’s life, may predict attributions of emotion and cognition. Although cat caregivers describe their companion animals primarily as family members (Bouma et al.) [31], an even greater proportion of dog owners describe their dogs as family members (Arahori et al.) [17]. The nature of the companion animal–human bond was a stronger mediator of the association between anthropomorphism of traits and relationship outcomes (i.e., making-up, social support, communication) for cats compared to dogs (Bouma et al.) [1], perhaps due to greater variation in the ascribed roles of cats. Bouma et al. [31] found that caregivers who described their cat as a child or best friend saw their cat as loyal and empathetic. In a later study, Bouma et al. [2] showed that owners who described the relationship with their cat in human terms (family member, best friend, or child) more often assigned complex social emotions (such as jealousy and compassion) to cats. Similarly, Arahori et al. [17] found that cat owners who described their cats as family members were more likely to attribute “compassion” to their cats compared to owners who did not describe their cats as family members. 

We did not specifically focus on the role of the cat here, as we assumed that the majority of cat caregivers would describe their cat as a family member or companion. However, we did ask about housing conditions, specifically whether the cat was housed exclusively indoors or outdoors or permitted access to both indoor and outdoor spaces. Cats that spend more time indoors are likely to spend more time with their caregivers, giving caregivers greater opportunities to observe their cognitive abilities and emotions. Thus, it is important to measure both housing conditions or time spent with caregivers and attachment to potentially tease apart the unique role of attachment. Given research showing associations between housing conditions and owner attachment, grief (e.g., [32]), and cat cognition [33], we anticipated that housing conditions might predict attributions of emotional and cognitive abilities. Our study builds on previous research to allow for an examination of the complex associations between cat ownership, attachment strength and style, housing conditions, beliefs in animal minds, and attitudes toward animals as predictors of primary and secondary emotions and cognition.

## 2. Method

This study was reviewed and approved by Oakland University’s Institutional Review Board (#2022-56).

## 3. Participants

We recruited 489 students enrolled in an introductory psychology course at a Midwestern University in the United States. We eliminated data from 41 students who failed an attention check or had incomplete data, leaving us with valid data from 448 participants, 251 of whom had owned a cat previously (*n* = 7) or at the time of the study (*n* = 244). Of the cat owners, whose average age was 20.19 years (SD = 3.24), there were 197 females, 45 males, and 9 participants who identified as non-binary. They had been cat owners for a total of 3.88 years on average (SD = 1.25). Of the non-cat owners, whose average age was 19.68 (SD = 2.22), 146 were female, 48 were male, and 3 identified as non-binary. Of the total sample, 324 were White, 50 were Black, 18 were Asian, 16 were Hispanic, and 40 identified as “other”.

Participants completed the following measures online at a secure website, Qualtrics.com.

## 4. Materials

All participants answered demographic questions to indicate their gender, age, and ethnicity. Participants also indicated what kind of pets they currently owned and had owned previously, as well as the number of pets of each kind. Only those who indicated having been cat owners at any point answered questions about whether the cat was kept mostly indoors or outdoors. They also answered a number of questions specific to the experience of jealousy in cats, which were not reported here because these questions were meant to inform the design of a future study. 

Participants answered the extent to which they felt that cats could experience various emotions (fear, anger, sadness, disgust, surprise, joy, interest, lust, love, grief, guilt, gratitude, shame, envy, pride, pity, self-pity, admiration, humility, embarrassment/humiliation). These emotions were the same as those included in Bouma et al.’s [1] comparative anthropomorphism measure (COANT), with the exception that we included humility, disgust, envy, self-pity, lust, and interest, and [1] additionally included care and jealousy. Our secondary emotions also differed from those reported on by participants in other previous studies (i.e., shame, jealousy, disappointment, and compassion for [5]; shame, jealousy, regret, and compassion for [2]). We used the same scale used by [1], with the exception that participants rated the emotions on a 7-point scale (instead of an 8-point scale) ranging from “1 = No. Cats cannot experience this emotion” to “7 = Yes, in a way that is totally comparable to humans”.

Participants also rated the extent to which they felt that cats were capable of 27 behaviors and cognitive traits intended to represent both social and non-social cognition, in which social cognition involved reasoning about other agents. This scale was similar to the COANT measure used by [1] but included a broader range of cognitive abilities. Bouma et al.’s items could also be classified as capturing social (understanding human emotions, understanding human language, judging someone, deceiving someone on purpose, and taking revenge) and non-social (reasoning, counting, making decisions, thinking about the future, and thinking about the past) cognition. Here, we used a 7-point rating scale from “1 = No. Cats are not at all capable of this” to “7 = Yes, in a way that is totally comparable to what humans are capable of”, whereas Bouma et al. used an 8-point scale with the same endpoints. 

### 4.1. Belief in Animal Minds

The Belief in Animal Minds Scale (BAM, [28]) includes four items: “Most animals are able to think to some extent to solve problems and make decisions about what to do”. Participants used a 7-point scale of “1 = strongly disagree” to “7 = strongly agree”, α = 0.619.

### 4.2. Pet Attitude Scale

We used the modified version of the Pet Attitude Scale (PAS; [29]), which contained 18 items assessing participants’ positive and negative thoughts about pets, such as “I really like seeing pets enjoy their food”. Participants used a 7-point scale (1 = “strongly disagree” to 7 = “strongly agree”), α = 0.836. Negative items were reverse-scored, such that higher scores indicated more positive attitudes.

### 4.3. Censhare Pet Attachment Scale

Only those participants who had owned a cat responded to this scale, and they responded with regard to the cat that they felt most attached to. The CENSHARE Pet Attachment Scale [34] includes 27 items, such as “You confide in your pet”. Participants respond to these items using a 4-point Likert scale (0 = “almost never”, 4 = “almost always”). Inter-item reliability was high; α = 0.929.

### 4.4. Companion Animal Bonding Scale (CABS)

The Companion Animal Bonding Scale [35] included eight items, such as “How often were you responsible for your companion animal’s care?” Participants responded on a 5-point scale where 1 = “never” and 5 = “always”, α = 0.866. Only those participants who had owned a cat responded to this scale, and they responded with regard to the cat that they felt most attached to. 

We averaged the standardized scores on the CENSHARE and the CABS to create a single measure of attachment strength.

### 4.5. Pet Attachment Questionnaire

The Pet Attachment Questionnaire [9] includes two subscales, each containing 13 items, to assess avoidant (α = 0.924) and anxious attachment (α = 0.866). An example item for avoidant attachment is “I try to avoid getting too close to my pet”. An example item for anxious attachment is “I need a lot of reassurance from my pet that it loves me”. Participants responded using a 7-point scale where 1 = “strongly disagree” and 7 = “strongly agree”. Only those participants who had owned a cat responded to this scale, and they responded with regard to the cat that they felt most attached to.

## 5. Results

First, we conducted two separate principal component analyses to explore the factor structure of the emotions and the cognitive skills attributed to cats. We applied an oblique ProMax rotation, restricted eigenvalues to >1.0, and considered items to belong to components with factor loadings > 0.45. Three components were extracted for emotions (Table 1). The primary emotions, along with envy, interest, grief, and love, were most strongly loaded onto Factor 1, with guilt, gratitude, admiration, shame, pity, humility, and embarrassment/humiliation loading on to Factor 2, and lust, self-pity, and pride loading on to Factor 3. Because these last three items cross-loaded onto Factor 2 with factor loadings > 0.46, we combined emotions from Factors 2 and 3 together into the category of secondary emotions. Notably, envy was the only emotion we had described as secondary that loaded more strongly with the primary emotions, so we included it with the primary emotions.

For cognition, although five factors were extracted, all items (except for “form attachments to humans”) loaded most strongly on Factor 1, which accounted for 40.54% of the total variance (Table 2). Given the large drop-off in eigenvalues after Factor 1 (from 31.68 to 6.19), we analyzed the cognitive skills together as a single measure of cognition rather than distinguishing social and non-social cognition as initially planned.

Descriptive statistics and correlations appear in Table 3. Independent sample t-tests revealed significant differences between cat caregivers and non-caregivers, with cat caregivers having more positive attitudes toward pets (*t*_347.94_ = −4.383, *p* < 0.001, 95% CI: −0.585, −0.223) and belief in animal minds (*t*_380.27_ = −2.606, *p* = 0.010, 95% CI: −0.454, −0.063). Caregivers also attributed significantly more primary (*t*_384.05_ = −2.977, *p* = 0.003, 95% CI: −0.523, −0.107) but not secondary emotions or cognition compared to non-caregivers. Equal variances were not assumed. 

We regressed the outcomes (primary emotions, secondary emotions, and cognition) on the predictors (caregiver status, attitudes toward animals, and belief in animal minds) for the entire sample. Examination of VIFs suggested that multicollinearity was not an issue. We applied a Bonferroni correction and applied an alpha of 0.02 because we conducted three regressions for each set of predictors. Positive attitudes (*β* = 0.227, *t* = 4.613, *p* < 0.001, 95% CI: 0.142, 0.352) and beliefs in animal minds (*β* = 0.316, *t* = 6.481, *p* < 0.001, 95% CI: 0.222, 0.416) were associated with attributions of primary emotion. Only beliefs in animal minds predicted attributions of secondary emotions (*β* = 0.178, *t* = 3.269, *p* < 0.001, 95% CI: 0.072, 0.290) and cognition (*β* = 0.250, *t* = 4.610, *p* < 0.001, 95% CI: 0.145, 0.360).

We also regressed the same outcomes—primary emotions, secondary emotions, and cognition—on the predictors: attachment strength (which was a composite measure of the average of the CENSHARE and CABS scales), avoidant attachment, anxious attachment, and housing status (indoors/outdoors) for the subset of the sample that were caregivers. Housing was categorized dichotomously as primarily indoors or primarily outdoors. Only avoidant attachment negatively predicted the attribution of primary emotions (β = −0.216, *t* = −2.605, *p* = 0.010, 95% CI: −0.403, −0.056). Only attachment strength predicted attribution of secondary emotions (β = 0.200, *t* = 2.336, *p* = 0.020, 95% CI: 0.038, 0.451) and cognition (*β* = 0.252, *t* = 2.910, *p* = 0.004, 95% CI: 0.102, 0.530). 

## 6. Discussion

Based on previous research [3,4,5,6], we anticipated that participants with greater attachment strength and less maladaptive (avoidant or anxious) attachment styles would be more likely to attribute complex emotions and cognitions to their pet cats. This hypothesis was somewhat supported by the finding that cat caregivers with avoidant attachment styles were less likely to attribute primary emotions to their cats, but there were no other associations with avoidant attachment and none with anxious attachment. Attachment strength, but not attachment style, predicted the attribution of secondary emotions and cognition as expected. Companion animal caregivers are more likely to attribute primary rather than secondary emotions to their animals [3,4], although [19] found high levels of attributions of jealousy and [5] found high levels of compassion being ascribed to companion animals. Nonetheless, there may be more variability in attributions of secondary emotions and, thus, greater opportunity to observe the effects of caregiver attributes. For example, Walker et al. [36] found that men and women did not differ in the attribution of primary emotions to animals, but women were more likely than men to ascribe grief for a separated companion to companion animals.

Notably, the general pattern of our results is consistent with previous findings, despite some discrepancies in the specific traits and emotions that respondents reported and different methods of scoring their responses. For example, our scoring of attributions allowed a greater range of responses compared to some previous research; Su and Martens [5] used a 3-point scale for participants to indicate whether their animal had ever expressed the given emotion (never, sometimes, often), and [3] asked owners to simply indicate whether they had witnessed these emotions in their animals using a dichotomous response (no, yes). Arahori et al. [17] asked more general questions, such as whether owners thought their animals had intelligence, reason, and could read attentional states and intentions using a 5-point scale, and separated items into emotional or intellectual items. The replication of findings that greater attachment predicts greater attributions of emotion and cognition despite these methodological differences points to the robustness of the findings.

Furthermore, previous studies asked participants to what extent their animals were capable of expressing primary and secondary emotions [3,4,14] or different categories of emotion [1,2], but have not asked to what extent animals are capable of attributing those emotions to others, which would likely reveal greater species differences and perhaps stronger impacts of caregiver attachment. We asked whether cats were capable of understanding the emotions of other cats and humans. These responses were included in our score for cognition. Our principal component analysis revealed that the 27 cognitive items could be conceptualized as representing a single cognitive factor; however, there may be interesting differences in the ascription of specific cognitive abilities if examined independently. Vonk [6] asked dog and cat caregivers to rate how well their companion animal responded to their name, followed points/gestures, provided comfort to other animals, provided comfort to owners, learned new routines/behaviors, solved problems, anticipated the actions of others, anticipated the outcomes of its own actions, explored novel objects, and engaged in hunting behaviors. Pairs of these items were combined to create the outcomes: point/name, learn/solve, hunt/explore, comfort, and anticipate. Attachment strength predicted greater attributions of skill for each of these categories of cognitive skill, so it is possible that our results reflect the fact that caregivers with strong, secure attachments are simply more likely to attribute cognitive skills of all types to their companion cats compared to those with less strong or secure attachments. It is also possible that more strongly attached caregivers are more sensitive to their cats’ psychology and are better positioned to observe their abilities compared to those with less secure attachment styles. 

It is important to note that, although attachment strength predicted attribution of secondary emotions and cognition, cat caregivers were not more likely to attribute emotions or cognition to cats compared to non-cat caregivers, which somewhat weakens the idea that experience caring for animals leads to greater attribution of their abilities. This is despite the fact that caregivers had more positive attitudes toward cats and greater beliefs in animal minds. Most previous studies have focused only on animal caregivers, with a stronger focus on dog than cat caregivers, and have not compared their beliefs to those of non-animal caregivers, although see [23], who found that companion animal caregivers were more likely, compared to non-caregivers, to attribute grief to their animals when they were separated from a conspecific. 

We also hypothesized that non-cat caregivers who had more positive attitudes toward animals in general and those who had stronger beliefs in animal minds would attribute more complex emotions and cognitions to cats. However, whereas both predictors were associated with the attribution of primary emotions, only beliefs in animal minds predicted attributions of secondary emotions and cognition. This finding is expected because belief in animal minds represents a general belief that animals are capable of human-like feelings and are conscious, intentional agents. Although this finding may be considered somewhat circular, it indicates that these general beliefs about animal minds apply specifically to companion cats. This is important because animal caregivers tend to anthropomorphize cats less than dogs [1,13] and attribute fewer complex cognitions to them [1,6,13,14,17,18]. It is of interest that positive attitudes toward animals [29], which reflect a fondness or liking for animals rather than beliefs in animals as being intelligent, predicted only primary emotions but not more complex emotions or cognition. This finding suggests that people need not attribute high levels of cognitive ability to animals to appreciate them. 

We also studied the impact of housing, as we expected that caregivers would be more likely to attribute emotional and cognitive complexity to companion animals that were more closely integrated with the daily lives of their human caregivers and spent more time indoors. Although keeping companion animals indoors was associated with greater attachment strength, beliefs in animal minds, more positive attitudes about animals, and lower levels of avoidant attachment, it was only weakly correlated with an understanding of primary emotions and was not correlated with our other outcomes. It was not a unique predictor of any of our outcomes. Thus, if housing has an impact, as it has had in other studies examining companion animal/caregiver interactions (e.g., [32]), it may be mediated by higher levels of attachment to indoor animals and different beliefs about animal minds in general. Jordan and Vonk [32] found that caregivers reported less attachment to animals that were enclosed compared to those that were able to roam freely, but they did not distinguish between free-roaming indoors or outdoors (due to the fact that they were primarily interested in exotic pets that did not have the capacity to roam freely out of the home). However, at least one study found that people who kept dogs indoors reported higher levels of attachment compared to those who let their dogs outside for longer periods of time [37]. In addition, Bouma et al. [31] showed that owners who let their cats roam outside were less likely to perceive their cats as children, although this might be related to a higher likelihood of these owners having pedigree cats. Thus, the impact of housing may be difficult to disentangle from the impact of attachment.

Our respondents were fairly generous in their attribution of emotional and cognitive complexity to cats. Although we largely replicated and extended previous findings, it should be noted that there were some differences in how we framed our measures compared to previous work. For example, we asked our participants the extent to which they thought that cats, in general, were capable of expressing different emotions (as in [1]), whereas other researchers [5] asked caregivers explicitly whether they had observed their companion animals expressing given emotions. If anything, our framing might be expected to lead to an underestimation of the attribution of cat abilities because caregivers might be most likely to attribute emotions to an animal they are bonded with. 

## 7. Limitations

Although our focus was not on anthropomorphism per se (like [1]), we used the same scale endpoints for the questions about cognitive and emotional abilities, which forced respondents to indicate how much they perceived their companion animal’s abilities to be similar to those of humans. If respondents thought that the animal had the ability but in a form that was different from how it is expressed in humans, they would have assigned an intermediate score of “yes, but not to the same degree that humans are”, which may not reflect accurately scaled estimates of the degree of complexity of the animal’s understanding. In future studies, we would use a more animal-centric scale without explicit reference to humans’ abilities. 

Although we examined the role of housing conditions (indoor and outdoor access), it would be of greater interest to focus on rearing experiences in terms of early socialization and how those experiences are associated with later cognitive and emotional outcomes. Vonk [6] attempted to examine these trends in a previous study, but most companion animal caregivers had adopted cats and dogs post-rearing, and thus, many did not know their animals’ early experiences. Furthermore, animals that are relatively unsocialized to humans may be less likely to have caregivers that participate in these surveys, restricting the range of this variable. However, Vonk [6] did show some effects of early exposure to humans on both attributions of emotions expressed and cognitive capacities, like the ability to follow points and attend to names, at least for dogs. In addition, that study revealed the effects of time spent together, which are probably only partially captured by our measures of attachment. However, the more time spent together, the more opportunities exist for observing interesting facets of companion animal behavior, so this predictor should be controlled in future studies.

Our sample was not representative of cat caregivers in general, as we surveyed only college students in the U.S. Because these participants all had at least some college education and also because they were enrolled in a psychology class, they were probably more likely to attribute intelligence to other species compared to the general public, which may be less well educated about animal cognition. It is also true that those who voluntarily completed our survey were likely more interested in animal cognition than those who opted not to participate. Therefore, we may have captured limited variability in both our predictors and outcomes concerning cat cognition. However, it is worth noting that [1] noted similar limitations but still found quite a bit of variability in their measure of anthropomorphism and noted that this was also true for other studies [3,17]. Our sample was predominantly White and female, so the results may not generalize to other groups, especially individuals in less developed countries who may not have the same understanding of animal welfare [5]. It is notable that prior studies involved non-American samples, e.g., British [3,14], Chinese [5], Japanese [4,17], Romanian [13], and Dutch [1,2]. Despite the cultural differences in attitudes about companion animals revealed by prior studies [11,38,39], the similarities of our results to previous studies reveal common associations between attachment and attributions of psychological traits in nonhumans. We did not ask about religious beliefs, which may be associated with different beliefs about animal minds, and that would be an interesting direction for future work. 

Most critically, our study focused on caregiver perceptions of animal abilities, but we cannot speak to whether their animals actually differed in ability because no actual tests or observations were performed on the animals. Thus, we cannot differentiate between hypotheses about whether owners simply perceive their animals to be more emotionally and cognitively complex when they are more strongly attached to their animals or whether owners may develop stronger attachments to animals that they believe to express more complex emotions or cognitions. Controlling for the actual abilities of the cats would be ideal for future studies to determine whether such associations reflect bias or actual differences, but it would be challenging to implement with such large sample sizes. 

## 8. Conclusions

Despite these limitations, the current study adds to the growing literature showing how caregivers’ attachments to their pets may shape their thoughts about companion animal minds. Alternatively, it could be that companion animal caregivers with high levels of secure attachment to their animal companions have greater insight into their emotional and mental lives compared to those with weaker attachments or a lack of caregiving experience. Because anthropomorphizing animals has been linked to greater empathy for animals [13,40] and stronger social connections between companion animal caregivers and their animals [1], but also to greater errors in interpreting the emotions of their cats [2], it is important to understand the factors predicting these attributions to maximize animal welfare. 

## Figures and Tables

**Table 1:** Rescaled Components and Factor Loadings for Emotions.

Emotion	Factor 1 (Primary)	Factor 2	Factor 3
Fear	0.764	0.274	0.151
Anger	0.716	0.377	0.424
Sadness	0.785	0.512	0.275
Disgust	0.549	0.442	0.439
Surprise	0.667	0.295	0.458
Joy	0.740	0.428	0.245
Interest	0.691	0.296	0.268
Love	0.672	0.411	0.343
Envy	0.670	0.382	0.365
Grief	0.725	0.590	0.378
Guilt	0.529	0.853	0.387
Gratitude	0.547	0.711	0.439
Shame	0.488	0.855	0.349
Admiration	0.568	0.673	0.496
Pity	0.405	0.829	0.532
Embarrassment	0.288	0.750	0.626
Humility	0.282	0.832	0.626
Self-pity	0.239	0.671	0.802
Pride	0.543	0.455	0.787
Lust	0.405	0.518	0.755
% Variance			

**Table 2:** Rescaled Components and Factor Loadings for Cognition.

Cognition	Factor 1	Factor 2	Factor 3	Factor 4	Factor 5
Cheat	0.599	0.025	−0.404	0.022	0.349
Deceive	0.592	0.416	−0.242	0.070	0.277
Judge others	0.645	0.359	−0.376	0.011	−0.194
Get revenge	0.595	0.453	−0.406	0.010	−0.204
Behave spitefully	0.604	0.500	−0.228	0.039	−0.136
Understand other cats’ emotions	0.599	0.320	0.282	0.163	−0.057
Understand human emotions	0.549	0.201	0.323	0.223	−0.077
Recognize their own reflection	0.571	−0.047	0.119	0.330	−0.045
Understand human language	0.604	−0.136	0.306	0.391	−0.039
Understand others’ intentions	0.679	−0.066	0.115	0.407	−0.068
Understand what others see	0.671	−0.267	0.006	0.376	−0.175
Understand what others know	0.659	−0.391	−0.149	0.309	−0.083
Understand death	0.639	−0.270	0.109	−0.188	−0.134
Understand human points and gestures	0.613	0.184	0.427	−0.096	0.026
Manipulate human behavior	0.692	0.233	0.201	−0.321	0.119
Manipulate the behavior of other animals	0.711	0.175	0.204	−0.339	0.070
Think about their own knowledge	0.730	−0.282	−0.074	−0.234	−0.316
Think about their own existence	0.661	−0.293	−0.113	−0.240	−0.357
Form friendships with animals	0.577	0.214	0.441	−0.095	0.009
Form attachments to humans	0.383	0.311	0.501	−0.031	−0.004
Count	0.659	−0.273	−0.228	−0.012	0.307
Solve complex problems	0.662	−0.198	−0.118	−0.002	0.423
Use objects as tools	0.603	−0.233	−0.001	0.045	0.361
Make conscious decisions	0.717	0.002	0.072	0.089	0.141
Think about the past	0.685	−0.336	−0.088	−0.141	−0.107
Plan for the future	0.676	−0.380	−0.185	−0.147	−0.033
Think about absent objects	0.652	−0.136	0.155	−0.275	0.086
% Variance	40.55	7.92	6.51	4.57	4.04

**Table 3:** Descriptive Statistics and Correlations for the Key Variables.

	1	2	3	4	5	6	7	8	9	10
1. Caregiver	--									
2. Housing	0.11	--								
3. Attachment	0.15 **	0.22 ***	--							
4. Avoidant	−0.18 ***	−0.13 *	−0.58 ***	--						
5. Anxious	−0.02	0.00	0.30 ***	0.30 ***	--					
6. Attitudes	0.20 ***	0.23 ***	0.70 ***	−0.70 ***	0.01	--				
7. Beliefs	0.12 **	0.22 ***	0.35 ***	−0.43 ***	−0.11 *	0.51 ***	--			
8. Primary Emotions	0.14 **	0.14 *	0.31 ***	−0.31 ***	0.03	0.40 ***	0.44 ***	--		
9. Secondary Emotions	0.03	−0.06	0.18 ***	−0.00	0.08	0.14 **	0.20 ***	0.65 ***	--	
10. Cognition	0.01	0.03	0.19 ***	−0.00	0.12 **	0.14 **	0.26 **	0.59 ***	0.64 ***	--
Mean	0.56	0.91	0.02	1.94	3.08	5.59	5.35	5.24	3.60	3.74
SD	0.50	0.28	0.92	1.02	1.03	0.81	0.94	1.09	1.36	1.07

Note. * *p* < 0.05, ** *p* < 0.01, *** *p* < 0.001.

## Data Availability

Further inquiries can be directed to the corresponding author(s).
